# Supplementary material for: Experimental evaluation of genomic DNA degradation rates for the pathogen Pseudogymnoascus destructans (Pd) in bat guano
Source: PeerJ. 2020 Jan 20;8:e8141. doi: 10.7717/peerj.8141 (PMC6977466; doi:10.7717/peerj.8141)
Supplement: Table S1 [file peerj-08-8141-s001.docx]

**Supplementary Table 1***.*

Model output with estimated regression parameters, standard error, z-values and P-values for the binomial GLM presented in equation 1 to explain the probability of detecting positive samples of genomic DNA of *Pseudogymnoascus destructans*

Model

glm(formula = positive ~ quantity.x + newsample * site.x + mass, family = "binomial", data = df_nazero)

Deviance Residuals:

Min 1Q Median 3Q Max

-3.08602 -0.29223 0.08844 0.29571 2.55728

|  | **Estimate** | **Std. Error** | **z value** | **P-value** |
| --- | --- | --- | --- | --- |
| Intercept | 3.97140 | 0.66695 | 5.955 | 2.61e-09 *** |
| 100,000 fg | -0.70515 | 0.34290 | -2.056 | 0.039743 * |
| 10,000 fg | -3.87914 | 0.38517 | -10.071 | < 2e-16 *** |
| 1,000 fg | -7.31125 | 0.50106 | -14.592 | < 2e-16 *** |
| Time | -0.01651 | 0.01084 | -1.524 | 0.127627 |
| Site Nisqually Site 2 | 4.00556 | 0.75004 | 5.340 | 9.27e-08 *** |
| Site Ohanapecosh Site 1 | 0.45666 | 0.65600 | 0.696 | 0.486344 |
| Site Ohanapecosh Site 2 | 1.64292 | 0.65936 | 2.492 | 0.012713 * |
| Site Carbon Site 1 | 0.62635 | 0.66286 | 0.945 | 0.344704 |
| Mass | 7.58537 | 1.95800 | 3.874 | 0.000107 *** |
| Days: Nisqually Site 2 | -0.10859 | 0.01636 | -6.637 | 3.20e-11 *** |
| Days: Ohana Site 1 | -0.09077 | 0.01660 | -5.470 | 4.51e-08 *** |
| Days: Ohana Site 2 | -0.08587 | 0.01543 | -5.564 | 2.64e-08 *** |
| Days: Carbon Site 1 | -0.01154 | 0.01500 | -0.770 | 0.441570 |

Signif. codes: 0 ‘***’ 0.001 ‘**’ 0.01 ‘*’ 0.05 ‘.’ 0.1 ‘ ’ 1

(Dispersion parameter for binomial family taken to be 1)

Null deviance: 1468.27 on 1076 degrees of freedom

Residual deviance: 571.31 on 1063 degrees of freedom

AIC: 599.31

**Supplementary Table 2** Model output with estimated regression parameters, standard error, t-values and P-values for the GLM presented in equation 2 to explain the amount of detected genomic DNA of *Pseudogymnoascus destructans.*

Model:

glm(formula = log(femto + 0.195) ~ quantity.x + newsample *

site.x + mass, data = df_nazero)

Deviance Residuals:

Min 1Q Median 3Q Max

-7.4211 -1.1102 0.2577 1.3470 5.5145

|  | **Estimate** | **Std. Error** | **t value** | **P-value** |
| --- | --- | --- | --- | --- |
| Intercept | 5.118749 | 0.282299 | 18.132 | < 2e-16 *** |
| 100,000 fg | -1.74982 | 0.153573 | -11.394 | < 2e-16 *** |
| 10,000 fg | -4.07240 | 0.154224 | -26.406 | < 2e-16 *** |
| 1,000 fg | -5.90272 | 0.153851 | -38.366 | < 2e-16 *** |
| Time | -0.01670 | 0.005187 | -3.221 | 0.00132 ** |
| Site Nisqually Site 2 | 1.449036 | 0.308535 | 4.697 | 2.99e-06 *** |
| Site Ohanapecosh Site 1 | 0.366183 | 0.309082 | 1.185 | 0.23638 |
| Site Ohanapecosh Site 2 | 1.255882 | 0.311144 | 4.036 | 5.82e-05 *** |
| Site Carbon Site | 0.059459 | 0.310883 | 0.191 | 0.84836 |
| Mass | 6.932004 | 0.890310 | 7.786 | 1.63e-14 *** |
| Time:Nisqually Site 2 | -0.049330 | 0.007265 | -6.790 | 1.86e-11 *** |
| Time:Ohanapecosh Site 1 | -0.064250 | 0.007246 | -8.868 | < 2e-16 *** |
| Time:Ohanapecosh Site 2 | -0.069759 | 0.007318 | -9.532 | < 2e-16 *** |
| Time:Carbon Site 1 | -0.013033 | 0.007276 | -1.791 | 0.07355 |

Signif. codes: 0 ‘***’ 0.001 ‘**’ 0.01 ‘*’ 0.05 ‘.’ 0.1 ‘ ’ 1

(Dispersion parameter for gaussian family taken to be 3.183902)

Null deviance: 12078.8 on 1076 degrees of freedom

Residual deviance: 3384.5 on 1063 degrees of freedom

AIC: 4319.6

Number of Fisher Scoring iterations: 2
